# A stochastic simulation model for assessing the masking effects of road noise for wildlife, outdoor recreation, and bioacoustic monitoring

**DOI:** 10.1007/s00442-022-05171-2

**Published:** 2022-05-06

**Authors:** Cory A. Toth, Benjamin P. Pauli, Christopher J. W. McClure, Clinton D. Francis, Peter Newman, Jesse R. Barber, Kurt Fristrup

**Affiliations:** 1 Department of Biological Sciences, Boise State University, Boise, ID 83725 USA; 2 Department of Biology, Saint Mary’s University of Minnesota, Winona, MN 55987 USA; 3 The Peregrine Fund, Boise, ID 83709 USA; 4 Department of Biological Sciences, California Polytechnic State University, San Luis Obispo, CA 93407 USA; 5 Department of Recreation, Park, and Tourism Management, Penn State University, College Station, PA 16802 USA; 6 Natural Sounds and Night Skies Division, National Park Service, Fort Collins, CO 80525 USA

**Keywords:** Anthropogenic noise, Coupled dynamics, Habitat quality, Road ecology, Soundscape

## Abstract

Traffic noise is one of the leading causes of reductions in animal abundances near roads. Acoustic masking of conspecific signals and adventitious cues is one mechanism that likely causes animals to abandon loud areas. However, masking effects can be difficult to document in situ and the effects of infrequent noise events may be impractical to study. Here, we present the Soundscapes model, a stochastic individual-based model that dynamically models the listening areas of animals searching for acoustic resources (“searchers"). The model also studies the masking effects of noise for human detections of the searchers. The model is set in a landscape adjacent to a road. Noise produced by vehicles traveling on that road is represented by calibrated spectra that vary with speed. Noise propagation is implemented using ISO-9613 procedures. We present demonstration simulations that quantify declines in searcher efficiency and human detection of searchers at relatively low traffic volumes, fewer than 50 vehicles per hour. Traffic noise is pervasive, and the Soundscapes model offers an extensible tool to study the effects of noise on bioacoustics monitoring, point-count surveys, the restorative value of natural soundscapes, and auditory performance in an ecological context.

**Supplementary Information:**

The online version contains supplementary material available at 10.1007/s00442-022-05171-2.

## Introduction

Anthropogenic noise levels have drastically increased over the past century (Slabbekoorn et al. 2010; Ortega 2012). In the continental USA, background sound levels have doubled in nearly two-thirds of all protected areas (Buxton et al. 2017). Roads are one of the most extensive human structures across the world (Forman et al. 2003) and their traffic generates a substantial amount of human-generated noise. Over 80% of the total land area is within 1 km of a road in the USA (Riitters and Wickham 2003), and more than 2.8 trillion vehicle miles were traveled in 2020 (http://www.fhwa.dot.gov/ohim/tvtw/tvtpage.cfm), despite the effects of the COVID-19 pandemic.

Diverse deleterious effects of noise are known, especially from roads (Barber et al. 2010; Kight and Swaddle 2011; Francis and Barber 2013; Shannon et al. 2016; Dominoni et al. 2020). Bird abundance or density often decreases with increasing noise levels along roadsides (e.g., Reijnen et al. 1996; Silva et al. 2012). These effects have been demonstrated for multiple taxa at relatively low traffic volumes (e.g., Forman et al. 2003; Charry and Jones 2009). Even some species that may be attracted to roadsides (e.g., to use as hunting grounds; Hindmarch et al. 2017) may suffer deleterious consequences from traffic noise (Mason et al. 2016; Senzaki et al. 2016). Decisive evidence comes from playback experiments that control for the presence of other confounding factors (e.g., mortality, chemical pollution, habitat fragmentation) associated with roads. One series of studies periodically broadcasted traffic noise at a migratory songbird stopover site; a 28% decline in bird abundance was found when noise was on, along with changes in age structure and a decline in body condition (McClure et al. 2013, 2017; Ware et al. 2015).

Though it seems clear that traffic noise plays a substantial role in the ecological impacts of roads, open questions remain concerning the ecological processes affected by noise and the quantitative relationships between noise exposures and responses. Also, there is substantial uncertainty regarding the effects of low traffic levels. Masking of important auditory cues is a significant problem (Brumm and Slabbekoorn 2005). A 3 dB increase in background sounds can reduce listening area by as much as 50% (Barber et al. 2010). The effects of masking have been documented up to 1 km from a noise source (Blickley and Patricelli 2012). Bird species that vocalize at low frequencies are masked more effectively by traffic noise and suffer the greatest reductions in proximity to roads (e.g., Goodwin and Shriver 2011; Francis 2015; but see Patricelli and Blickley 2006; Slabbekoorn and Ripmeester 2008). Traffic-noise exposure also can reduce hunting efficiency and success of acoustic predators (Siemers and Schaub 2011; Bunkley and Barber 2015; Senzaki et al. 2016). However, conclusive demonstration of auditory masking requires several pieces of physiological and environmental information (see Blickley and Patricelli 2010). Auditory detection thresholds are difficult to measure in free-ranging animals. Thus, distinguishing the effects of masking and other noise effects (Barber et al. 2010; Blickley and Patricelli 2010; Kight and Swaddle 2011; Dominoni et al. 2020) may prove intractable in field settings.

The noise emanating from roads depends upon traffic levels, vehicle characteristics, and driver behaviors (Subramani et al. 2012; Ramírez and Domínguez 2013). Road management practices can plausibly address each factor that affects total noise output. The dynamic interplay between spatiotemporal patterns of noise exposure and animal activities cannot readily be reduced to algebraic expressions, so simulations can be an important tool for understanding the most critical ecological aspects of noise exposure and informing effective noise control actions.

Noise also compromises human auditory experience in outdoor recreational settings. A growing body of work is exploring the coupled human–nature relationships associated with soundscapes in natural and urban settings (Francis et al. 2017). Anthropogenic noise not only displaces animals, but also interferes with the ability of humans or bioacoustic monitors to detect animals. Aural detection of wildlife is important for both psychological (Abbott et al. 2016; Ferraro et al. 2020) and scientific (Zwart et al. 2014; Koper et al. 2016) reasons. Accumulating research shows that anthropogenic noise diminishes the quality of visitor experience in natural areas, in part by masking natural sounds (Dumyahn and Pijanowski 2011; Stack et al. 2011; Rapoza et al. 2015). Noise also compromises acoustic wildlife surveys (e.g., Ortega and Francis 2012). On these grounds, it is important to include human dimensions with animal behavior in models of road noise effects and to recognize the potential for complex interactions in such models.

Individual-based models (IBMs) have emerged as a prominent tool for testing complex processes in many fields (Grimm et al. 2006), including ecology (e.g., Grimm et al. 1999; Grimm and Railsback 2005), because they grant researchers full control over experimental conditions. As their name implies, IBMs operate on the level of the individual, examining the emergent properties of populations based on the collective behaviors of individuals within a landscape and the effects of environments on those individuals. IBMs have been used to study how populations or communities respond to environmental stressors (e.g., Hall et al. 2006; Bennett et al. 2009; Beaudouin et al. 2012; Pauli et al. 2017), such as noise (e.g., Frankel et al. 2002; Nabe-Nielsen et al. 2014; Lacy et al. 2017) and roads (e.g., Ceia-Hasse et al. 2018).

Here, we present an IBM, called Soundscapes, to model the masking effects of traffic noise. It incorporates a standard model of noise propagation, calibrated spectra for vehicle noise output, and simple models for vehicle and searcher behavior. The concept of listening area plays a central role (see Box 2 in Barber et al. 2010). Specifically, the model assesses the ability of searching animals to locate acoustic resources when exposed to temporally dynamic road noise levels. The model also assesses the auditory detectability of these animals to human listeners, creating opportunities to explore coupled human-ecological dynamics in natural areas (Francis et al. 2017). Lastly, the model also assesses the performance of bioacoustic monitoring stations at detecting animals. The performance of alternative arrays of monitoring devices can be compared, including their sensitivities to noise, and the dependence of detections on animal density and behavior can be explored. We provide simulation results, illustrate Soundscape model features, describe potential applications, and outline opportunities for future elaboration of the model.

## Materials and methods

### The soundscapes model

We constructed the Soundscapes model (Online Appendix 1; https://github.com/kfristrup/SoundScapes) using NetLogo v6.2.1 (Wilensky 1999). Below we provide a concise description of the model. Online Appendix 2 provides a detailed User Guide and a description of the model using the updated Overview, Design concepts, and Details format (Grimm et al. 2006, 2010).

**Fig. 1:**
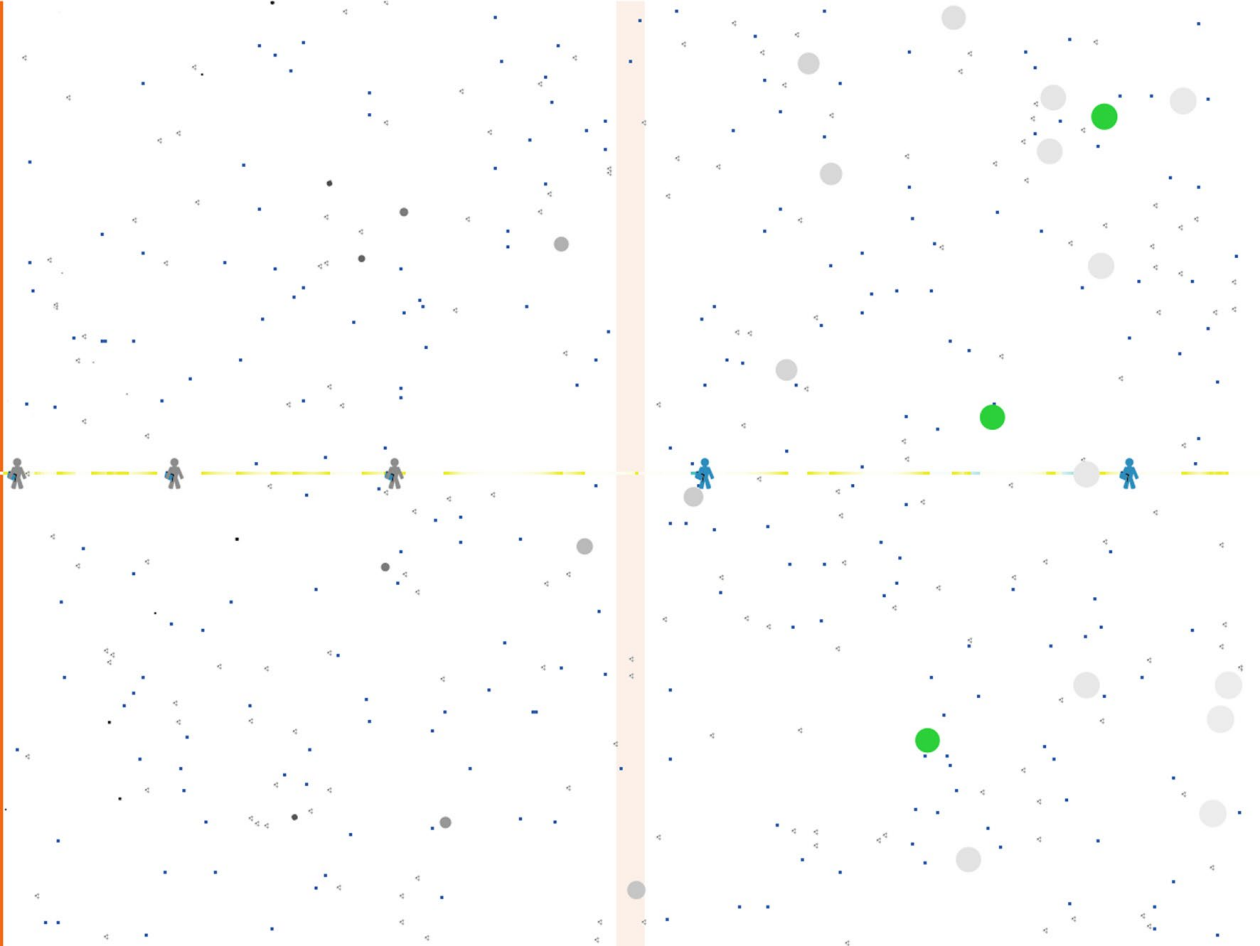
Soundscapes model showing a simulated landscape (400 × 301 pixels) affected by traffic noise. The left-most cells (i.e., *x* = 0) are designated as the road (*orange*) from which traffic noise propagates, while the centre horizontal cells (i.e., *y* = 0) are designated as a path (*yellow*). *Blue dots* represent resources placed randomly throughout the landscape. The listening area of searchers (i.e., the radius within which they can detect resources) is represented by the *grey circles*, the size of which decreases with increasing noise exposure in decibels. Searchers that are within perceptual range of a resource turn green. Stick figures represent listening stations (i.e., bioacoustic monitoring stations) placed semigeometrically along the trail. The trail and the stations turn blue when a searcher is within their perceptual range. A *yellow dot* on the trail marks that a searcher is in that column, but out of range. The *blue* and *yellow dots* on the trail fade toward the background color at a geometric rate, leaving “tails.” To achieve an indefinitely long road, landscape patches are also stacked column-wise in the landscape to form a continuous road that vehicles (seen as *grey dots*) travel along, top-to-bottom. The pale orange area (*x* ~ 200) denotes the road section within the landscape. Vehicles to the left of this area are moving toward the landscape, while those to the right are moving away. Vehicles exiting the top of the rightmost column reappear at the bottom of the leftmost column

Soundscapes forms a 400 × 301 pixel virtual landscape (hereafter, “the landscape”) with the left edge—and *y*-axis—designated as a *road*, and the *x*-axis running through the middle of the landscape as a *trail* (Fig. 1). Each pixel in the landscape represents 10 m × 10 m. When the model is initialized, some landscape cells are designated as stationary resources (see below). Resources are identical. Their distribution can be random or biased toward quieter areas. Resource density influences the tortuosity of searcher paths, but it has very little effect on the execution speed of the model. Another set of pixels is designated as *stations.* Stations might represent points of interest for human experience, animal survey locations, or bioacoustic monitoring stations. Stations require noise exposure computations; many stations could slow model execution.

The model has two mobile entities: *searchers* and *vehicles*. A user-specified number of searchers are distributed randomly throughout the landscape. Searchers are identical to one another, and represent animals that move within the landscape at each model iteration in search of resources. The speed and constancy of heading of searcher movement are set by the user.

The user also specifies the number and type of vehicles on the road. Vehicles are initially placed at random locations, though no two vehicles are placed on the same patch. All vehicles are initially traveling at their maximum speed. The road has a nominal speed limit, and each vehicle has a distinct maximum speed that differs from the speed limit by a random value drawn from a Gamma distribution. Vehicles accelerate at a constant rate until they reach their maximum speed or until they overtake a slower vehicle. Upon overtaking another vehicle each vehicle either passes without a change in speed or slows to a speed that is equal to or less than the vehicle in front of them. Excess deceleration creates an emergent property of bunched vehicles: congestion.

Resources are stationary and emit sounds continuously. Conceptually, these stimuli might be intentional signals (e.g., males signaling searching females) or adventitious sounds (e.g., prey cues; Goerlitz et al. 2008). The number of resources is specified by the expected distance between resource detections, assuming a straight course at the searcher’s speed and their maximum detection distance in the absence of noise. This setting, along with user-defined parameters for searcher movement speed and patterns, allow users to tailor the model to approximate the behavior of an animal of interest (e.g., a songbird searching for potential mates may travel further and with a straighter course than a foraging bat searching for insects). Searchers detect resources within their user-defined radius (Barber et al. 2010), which is inversely related to the sum of background sound and road noise levels. At each model iteration searchers move a constant distance on their present heading—which deviates from their previous heading by a Gaussian random variable—until they detect a resource they have not encountered previously. Upon detecting a resource, searchers move directly toward it until they land on the patch. Each resource is added to the searcher’s memory, and resources are never revisited.

All four edges of the landscape are impassable to searchers. Noise exposure is not symmetric along either axis, so allowing the landscape to “wrap” at either edge would introduce discontinuities in noise exposures when searchers traversed these edges. Searchers are reflected forward off each edge at a randomized heading that is biased away from the edge.

The listening area of humans on the centre trail and stations (which could be anywhere) are determine using a user-defined multiple of the searcher’s maximum detection distance. At each iteration the detection distance is reduced by an amount dependent on the level of noise exposure. During model initialization a noiseless “birdable probability” is calculated as the fraction of the total landscape area that is within the maximum detection distance (of searchers, by humans) of the central trail. While running, the model records the duration of time that searchers are detectable from the trail, and stations record the number of searchers that fall within their detection radius. Both resources and stations track the number of detections that would have occurred under noiseless conditions. Note that the ear-height of searchers, humans, and stations are all dictated by the same input parameter (*height-rcvr*), and thus are equal.

**Fig. 2:**
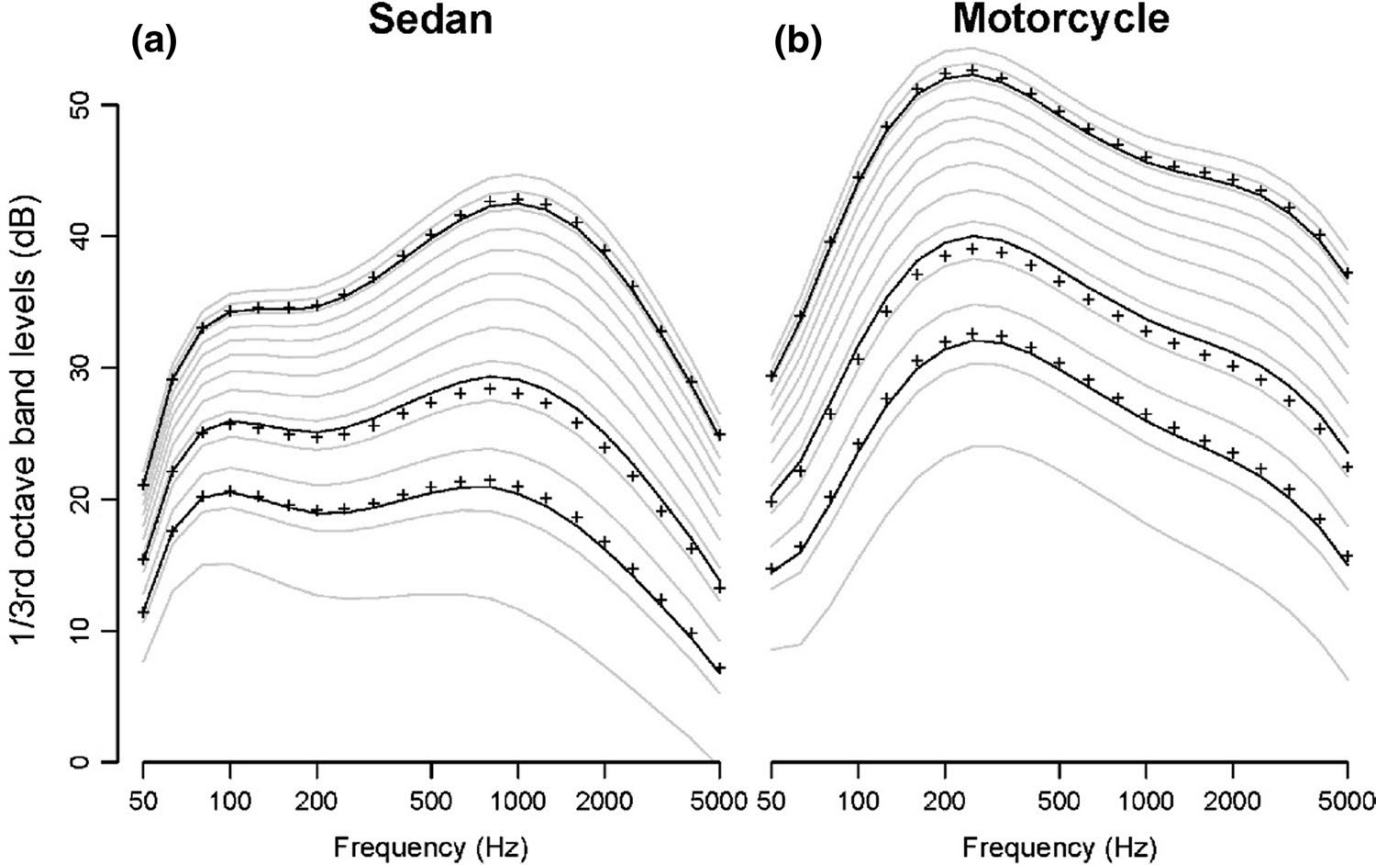
Power spectra across 1/3rd octave bands for Soundscapes’ two vehicle types: **a** sedans and **b** motorcycles. One-third octave band spectra at three speeds-6.7, 11.2, and 24.6 m/s, represented by *black markers*-were used to fit a power-law function for each spectral band from the NMSim noise model. *Grey lines* represent interpolated power spectra for additional speeds between 4 and 28 m/s in 2 m/s increments. *Black lines* denote fitted 1/3rd octave band levels at the reference speeds. Sedan noise output is most powerful around 1000 Hz, while motorcycle noise is both generally higher intensity overall and composed of much lower frequencies, with a peak around 250 Hz

Traffic noise in Soundscapes is generated from the integrated behavior of individual vehicles traveling on the road at varying speeds. The user selects between one of two vehicle types to populate the road prior to model initialization: sedans and motorcycles. The spectra and noise output of the two vehicles differ, with noise output of motorcycles being about tenfold higher than sedans (see below; Fig. 2). One consequence of these spectral differences is that motorcycle noise attenuates more slowly with distance. Vehicles within each class are identical. The level and spectral shape of their noise output changes with speed (the model does not account for increased engine noise while accelerating). The user specifies the hourly number of vehicles that pass the landscape, as well as the global speed limit of the road.

To achieve a road of acoustically “infinite” length, habitat patches were stacked column-wise to form a virtual road. This means that each patch has two sets of coordinates: road coordinates (patches are 1 m square) and ecological coordinates (patches are 10 m square). The road occurs at *x* = 0, and y coordinates range between ±200*301 + 150 m (60.35 km). The patches on this virtual road that correspond to the orange line in the ecological habitat are marked by pale orange in the central columns (*x* = 200; Fig. 1). Vehicles that are seen within the pale orange area are those that are currently traveling adjacent to the model’s landscape (i.e., the section of road denoted by the dark orange line at the model’s left edge). Columns to the left of this pale orange band represent road segments approaching the ecological habitat. Columns to the right represent departing road segments. Vehicles transiting beyond the top of one column reappear at the bottom of the column to the right. Vehicles at the bottom-left and top-right of the landscape and thus the furthest from the landscape. Vehicles exiting the top of the rightmost column reappear at the bottom of the leftmost column; functionally creating a road of indefinite length.

The user specifies atmospheric and other conditions that affect sound transmission, including ground hardness, temperature, and barometric pressure. An ambient background level for the landscape is also set by the user. This value is added to the incoming noise level to compute the total sound level that determines the reduction in acoustic detection distance. The propagation of traffic noise from the road follows the ISO 9613 calculations for both the absorption of sound by the atmosphere (ISO 9613-1; International Organization for Standardization 1993) and the attenuation of sound outdoors (ISO 9613-2; International Organization for Standardization 1996). Sedan and motorcycle noise output and spectra were drawn from 1/3rd octave band data packaged with the NMSim noise model (Wyle Laboratories and Blue Ridge Research and Consulting). One-third octave band spectra at three speeds were used to fit a power-law function for each spectral band (Fig. 2).

Model outputs in real-time include the average distance searchers need to travel to find a resource, both the noiseless birdable probability of the landscape (calculated at model initialization) and the actual birdable probability of the landscape, and traffic conditions (median vehicles·hour^−1^ and average speed). Additional outputs are available using BehaviorSpace processing (see Online Appendix 2). In the BehaviorSpace simulations presented below, the model iterated until a vehicle traveling at the speed limit would have traversed the road loop five times. For a full list of model parameters, see Table 1.

**Table 1:** Overview of input parameters of the Soundscapes model

Input parameters
*acceleration* The rate at which vehicles increase their speed in m·s^**−**2^
*ambient-level* The amplitude of the landscape’s ambient environment in dB
*atm-pressure-bar* The atmospheric pressure of the landscape in bar
*basePercept* The noiseless perceptual range (i.e., listening area) of searchers in meters
*calc-noise* A binary selection that determines if noise is calculated during model-run; turn off to rapidly simulate traffic dynamics and noise-free bioacoustic performance
*excess-brake* When an overtaking vehicle is unable to pass, it slows to the speed of the vehicle ahead minus excess-brake in kph. Larger values of excess-brake cause more congestion and stoppages in traffic flow
*ground-hardness* The hardness of the landscape’s terrain; lower values represent porous terrain (e.g., loose topsoil, powder snow) whereas higher values represent unyielding terrain (e.g., concrete)
*height-noiz* The effective height of the vehicle noise source in meters
*height-rcvr* The ear height of the receiver in meters
*human-mult* A multiplier for the size of the humans’ and listening stations’ listening areas relative to that of searchers’ noiseless perceptual range
*meters-moved-per-resource* The average distance a searcher would need to travel to encounter a resource under noiseless conditions; determines density of resources on the landscape
*noise-affects-resources?* A binary selection that determines if resources are distributed through random (off) or noise-constrained (i.e., weighted toward quieter areas; on) processes
*num-vehicles* The number of vehicles per model run.
*num-searchers* The number of searchers per model run
*relative-humidity* The relative air humidity of the landscape in %
*search-turn-sd* The standard deviation of differences between a searcher’s previous and next course heading, in degrees
*searcher-speed* The speed of searchers in m·s^**−**1^
*speed-limit* The global speed limit of the road in kph
*speed-limit-SD* The standard deviation of differences between individual vehicle maximum speeds and the global speed limit
*temp-celsius* The air temperature of the landscape in Celsius
*veh-name* A selection to choose the vehicle type.
*vpass-probability* The probability (from 0 to 1.0) that a vehicle will pass a vehicle in front of it

### Simulations and analyses

We performed 540 model runs for demonstration purposes. These consisted of 54 unique combinations of four input parameters: ambient sound level (*ambient-level*; 25, 30, 35 dB), speed limit (*speed-limit*; 40, 60, 100 kph), vehicle pass probability (*vpass-probability*; 0.1, 1.0), and number of vehicles per hour (*num-vehicles*; 5, 50, 500 per hour). We set atmospheric parameters to standard values for sea level (*atm-pressure*; 101 bar; *relative-humidity*: 60%; *temp-Celsius*: 20 °C) and we used an intermediate value for ground hardness (0.5). All runs used sedans for ease of comparison, and *excess-brake* was set to 5 km/h to generate traffic congestion when *vpass-probability* was 0.1. Each combination of parameters was simulated 10 times.

We varied two input parameters with ambient levels to increase comparability between runs. First, we decreased the base perceptual range of searchers (*base-percept*) via a logarithmic relationship with ambient sound levels: 50 m at 25 dB, 28.1 m at 30 dB, and 15.8 m at 35 dB. This was equivalent to assuming that the resource and searcher stimuli were at constant levels. Thus, we did not allow for the Lombard Effect, where callers increase their output to compensate for increased background sound levels (see Zollinger and Brumm 2011). These simulations also adjusted the *meters-moved-per-resource* in conjunction with ambient levels: 50 m at 25 dB, 88.9 m at 30 dB, and 158.1 m at 35 dB. Given the reductions in detection distance, these adjustments were equivalent to holding the spatial density of resources constant across the simulations.

To simplify portrayal of the output of the simulations, we grouped simulations that had similar—but not identical—vehicles per hour (VPH) traffic rates. We applied Sturges’ (1926) procedure to the log of VPH in each run. This resulted in eight bins of increasing traffic volume that we used as an index of noise exposure. All data generated during these runs are included in Online Appendix 3.

## Results

**Fig. 3:**
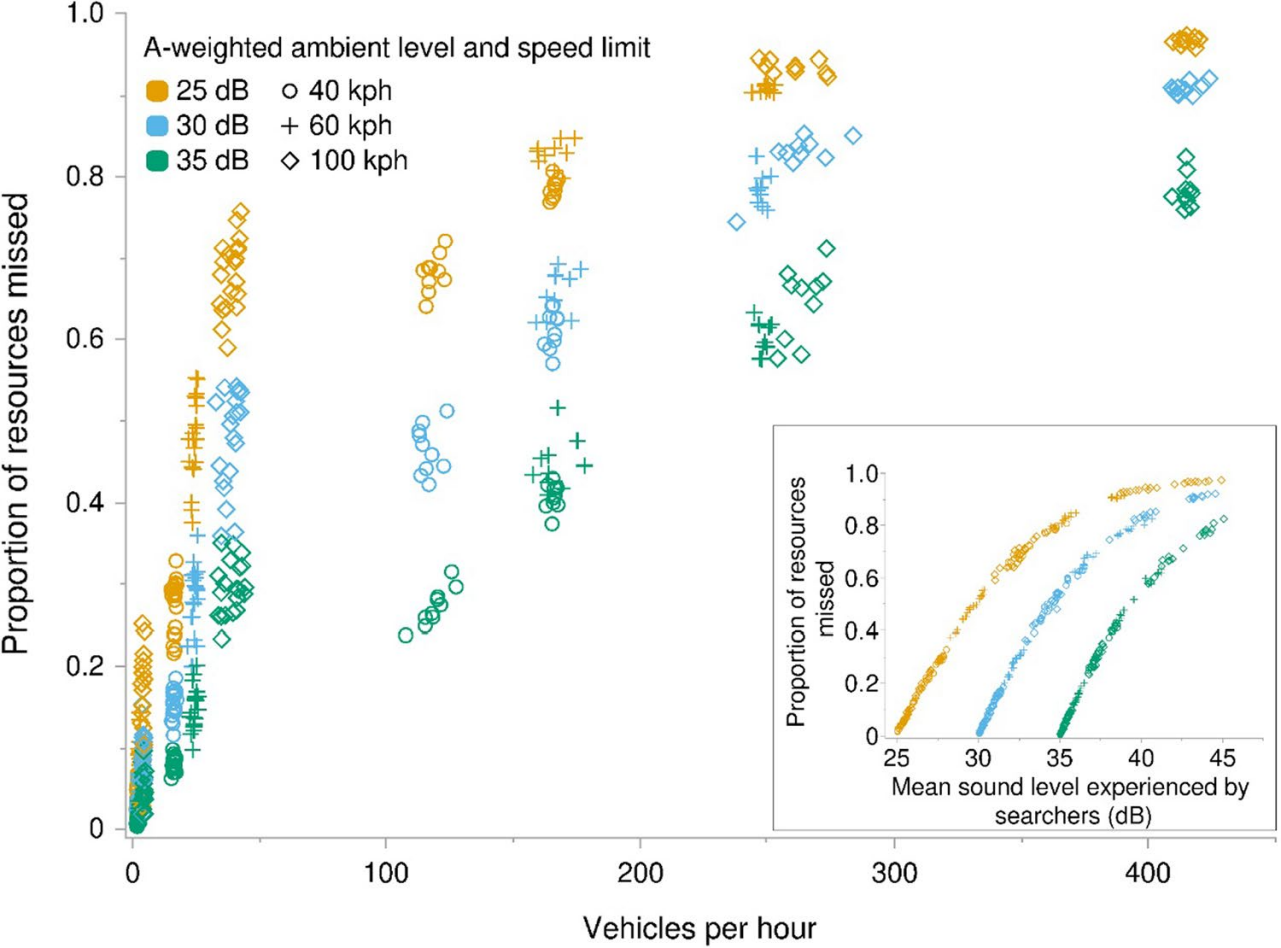
Proportion of acoustic resources missed by searching animals in a landscape adjacent to a road as a function of the number of vehicles that travel the road per hour and the mean A-weighted sound levels experienced by searchers during model runs (*inset*). Data generated from 540 model runs varying ambient sound level, speed limit, vehicle pass probability, and number of vehicles per hour. Each parameter combination was performed ten times. Vehicles per hour measures differ from input values as they represent actual traffic volume as influenced by varying maximum speeds among vehicles and congestion

**Fig. 4:**
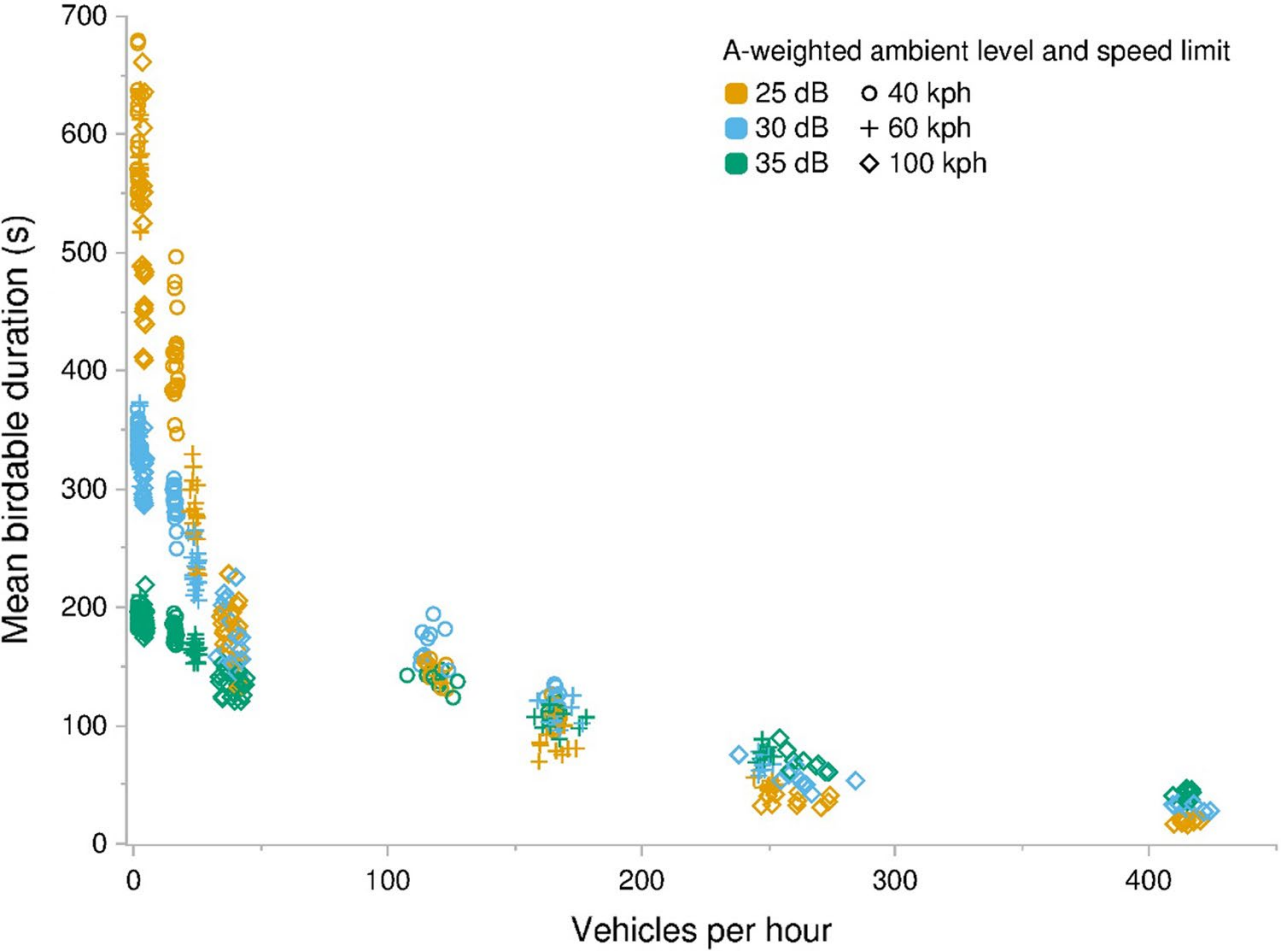
Mean duration that animals in a landscape adjacent to a road were audible from a trail running perpendicularly to the road, as a function of the number of vehicles that travel the road per hour. Data generated from 540 model runs varying ambient sound level, speed limit, vehicle pass probability, and number of vehicles per hour. Each parameter combination was performed ten times. Vehicles per hour measures differ from input values as they represent actual traffic volume as influenced by varying maximum speeds among vehicles and congestion

**Fig. 5:**
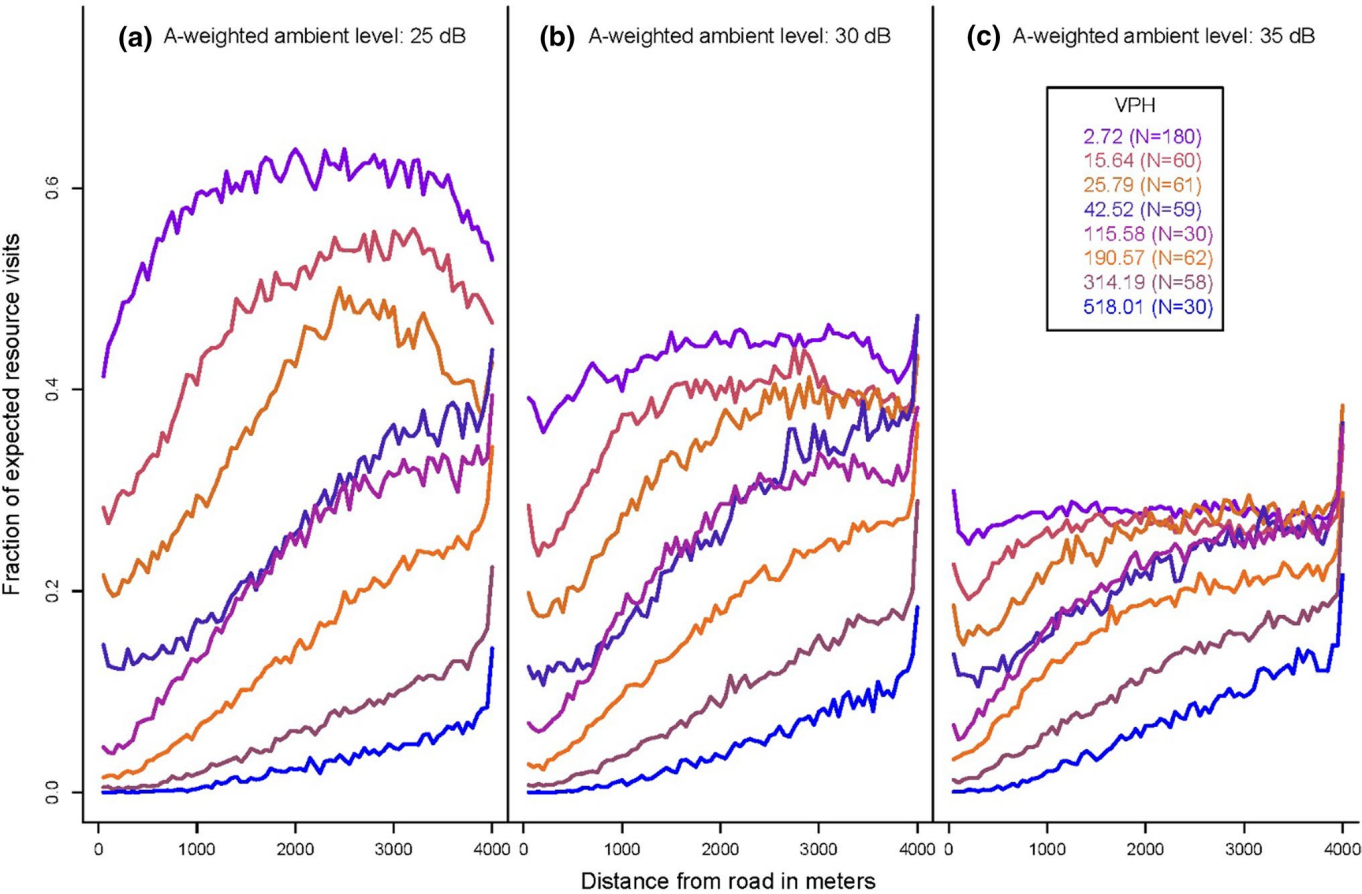
Fraction of total possible resource detections that were successful by searching animals as a function of distance from the road under A-weighted ambient levels of **a** 25 dB, **b** 30 dB, and **c** 35 dB. Data generated from 540 model runs varying ambient sound level, speed limit, vehicle pass probability, and number of vehicles per hour. Each parameter combination was performed ten times. Simulation data were grouped by Sturges binning of the log of vehicles per hour (VPH)

**Fig. 6:**
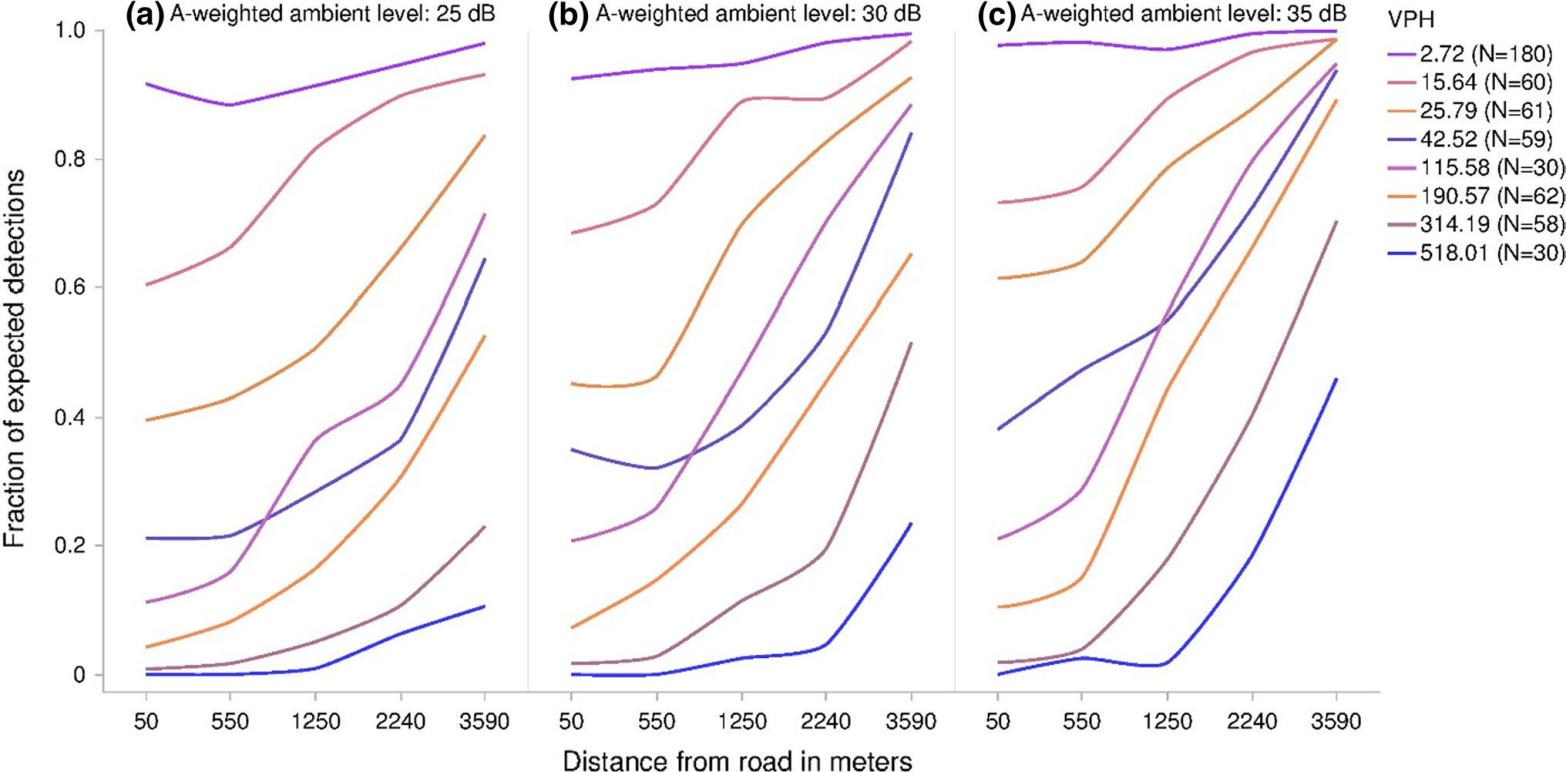
Fraction of total possible animal detections that were successful by five bioacoustic monitoring stations placed at semigeometric distances along a trail running perpendicularly to a road under A-weighted ambient levels of **a** 25 dB, **b** 30 dB, and **c** 35 dB. Data generated from 540 model runs varying ambient sound level, speed limit, vehicle pass probability, and number of vehicles per hour. Each parameter combination was performed ten times. Simulation data were grouped by Sturges binning of the log of vehicles per hour (VPH)

The ability of searchers to detect resources and the ability of humans and bioacoustic monitoring stations to detect searchers were all negatively impacted by increasing traffic noise levels, which were contingent on both traffic volume and speed. The proportion of acoustic resources that searchers missed increased with increasing traffic volume and with the level of noise each searcher experienced (Fig. 3). Similarly, the birdable duration of searchers decreased with increasing traffic volumes (Fig. 4). The effects of masking extended for considerable distances from the road. While the efficiency of searchers (Fig. 5) and listening stations (Fig. 6) generally increased with distance, even moderate traffic volumes impacted detections over a kilometer from the road’s edge.

Figure 5 reveals an artifact of the boundary effects on searcher movement. Resources that were located close to edges of the landscape were visited more often than resources located elsewhere. This artifact was caused by the forward reflection rule that governed searcher movements when they would otherwise have crossed the boundary. Our forward reflection rule had the benefit of reducing the overlap in search area along a reflected path. It introduced this artifact by increasing the number of searcher paths that approximately paralleled the landscape boundaries.

## Discussion

The Soundscapes model simulates the emergent properties that result from the masking effects of traffic noise and demonstrates the negative consequences for animals inhabiting spaces adjacent to roads, even under relatively low traffic volumes. Further, these masking effects extend to human users and bioacoustic monitoring stations within the landscape, decreasing the number of animals detectable within that landscape.

Our model quantifies a relationship between noise exposure and decreased acoustic search efficiency that likely contributes to patterns of decreasing animal abundances near roads. In our simulations, the onset of masking impacts for searchers could be observed under traffic volumes that correspond to roughly 240–480 vehicles per day, with more substantial effects at approximately 1200 vehicles per day under high vehicle speeds. This agrees with previous studies demonstrating the onset of effects for some taxa (e.g., birds, carnivores) at comparable traffic volumes (see Charry and Jones 2009). Such traffic volumes are not uncommon, and fall within the ranges of annual average daily traffic measures reported on rural roads throughout the United States (https://www.fhwa.dot.gov/policyinformation/travel_monitoring/pubs/aadt/). While other studies have predicted or observed impacts at low traffic volumes through mortality events (e.g., Gibbs and Shriver 2002, 2005) and road avoidance (e.g., Alexander et al. 2005), our model indicates that animals are also experiencing deleterious consequences from acoustic masking. These effects were pronounced in all of our simulations under higher ambient levels, and extended for the full extent of the landscape (4 km). Animals might be expected to abandon or avoid such areas completely under increasing traffic levels (e.g., Reijnen et al. 1995, 1996, 1997; Forman et al. 2002, 2003) due to the cumulative impacts of noise alone.

Human users of these landscapes were also affected by masking, as the duration that searchers were audible from a trail bisecting the landscape decreased as ambient level, traffic volume, and vehicle speed increased. Effects were particularly pronounced under high vehicle speeds with traffic volumes below 50 vehicles per hour, or roughly 1200 vehicles per day. These results highlight the coupling of natural and human systems, and the role soundscapes play in shaping human experiences in nature (Francis et al. 2017). Humans often seek natural areas to experience natural (biotic and abiotic) sounds (Marin et al. 2011), which in turn enhances user valuation of these spaces (Pilcher et al. 2009; Marin et al. 2011) and confers psychological benefits (Sandifer et al. 2015; Abbott et al. 2016). Transportation noise has become widespread in protected areas across the United States (Buxton et al. 2017), and our results suggest that reducing the amplitude of traffic noise could improve visitor experiences in natural areas through the reduction of acoustic masking of natural sounds (Levenhagen et al. 2020).

Similarly, simulated bioacoustic monitoring stations were negatively impacted, detecting fewer searchers closer to roads across all simulations. These effects were compounded under increasing traffic volumes, which could likewise extend several kilometers from the road’s edge. Extensive studies and surveys rely on the ability of human observers and bioacoustic monitoring stations to accurately detect acoustically signaling wildlife, particularly birds and amphibians (e.g., Bart 2005; Dodd Jr et al. 2012; Willacy et al. 2015; Shonfield and Bayne 2017). These have been shown to be affected even by moderately elevated background sound levels (e.g., Simons et al. 2007; Buxton and Jones 2012; Zwart et al. 2014), similar to our observed results. Simulations like these can be used to determine how many additional survey stations would be necessary to compensate for the effects of noise.

There are some caveats for interpreting the simulation results and parameter selection. For one, our assumption of continuous acoustic stimuli results in a masking effect that is expressed through reduced detection distance. For intermittent signals that are sparse enough that searchers would only experience one detection while passing by, the masking effect would be expressed through reduced listening area, which is proportional to the square of detection distance. For these signals, the decreases we documented would be shifted to lower traffic levels. For intermittent signals, gaps in traffic would have greater influence over detection probabilities, so we anticipate vehicle speeds and amounts of congestion will be more influential as stimulus presentation rates decrease.

Though A-weighted sums of sound energy are widely used to gauge noise effects in communities and ecosystems, it is widely acknowledged that this is a suboptimal measure for calculating the audibility of a sound. The Soundscapes model has an underlying spectral framework for characterizing noise output and sound propagation. Therefore, the model is prepared for an upgrade that would characterize the source spectra of the resource and searcher acoustic stimuli, and the auditory detection capabilities of searchers and human listeners. While this improvement was deemed out of the scope of the present effort, it would have the great benefit of opening additional simulation opportunities to explore the influence of spectral separation between vehicle noise and resource or searcher acoustic stimuli. Also, detection distance would no longer be an input parameter; it would be a result of the interaction between stimulus level and spectrum, ambient level and spectrum, and listener masked auditory performance.

Though we precompute noise propagation tables to speed model iteration, the present version may still be too slow for practical modeling when many hundred vehicles are simulated (although our results highlight that increased traffic volume beyond 400 vehicles per hour may not produce further insights for some outputs, without varying other input parameters). The product of the number of vehicles times the sum of the number of searchers and the number of stations offers the primary index of computational load per iteration. Were the model to be upgraded as suggested in the previous paragraph, this would slow iteration speed. Accordingly, it would be sensible to aggregate the noise propagation computations for groups of distant vehicles. It is probable that such aggregation could recover much of the simulation speed that would be lost by full spectrum calculations (instead of A-weighted calculations).

While we have provided a relatively narrow set of demonstrations, a strength of the model is the degree of customization afforded to users to generate several combinations of input parameters to suit their needs. Users can tailor simulations toward specific environmental conditions, traffic characteristics, or target animals. For example, road traffic has been identified as a significant challenge to the management of natural areas (e.g., Ament et al. 2008; Monz et al. 2016). Several options have been proposed to mitigate the effects of traffic noise in natural landscapes—such as communicating experiential expectations about soundscapes (Marin et al. 2011), or zoning approaches with clear management objectives (Stack et al. 2011)—that attempt to balance biodiversity/visitor experiences with site access (see Francis et al. 2017). Others include speed-limit reductions (Levenhagen et al. 2021) and reductions in traffic volume (e.g., through shuttle services or reservation systems). Use of the Soundscapes model can reveal which management options will be effective within their landscapes through simulation studies. The model can also reveal the potential negative impacts of noise under ranges of traffic conditions that might be challenging to document through field studies.

While Soundscapes provides a useful tool for researchers, managers, and planners to estimate or predict the negative consequences that may manifest under various traffic noise conditions, it does not replace the need for the monitoring of noise disturbance in situ. The calibrations built into this Soundscape model create opportunities to compare simulation outputs directly with field studies. These comparisons can cross-validate simulation results and offer a rigorous method for interpolating or extrapolation the field results. The increase in knowledge generated from such studies will be invaluable in developing efficient mitigation strategies that reduce the deleterious effects of traffic noise on human and non-human animals alike.

## Supplementary Information

Below is the link to the electronic supplementary material.

Supplementary file1 (RAR 33 KB)

Supplementary file2 (DOCX 355 KB)

Supplementary file3 (RAR 7936 KB)

## Data Availability

All data produced from this study are provided in this manuscript.

## Code availability

All source code for the model can be found in the GitHub repository at https://github.com/kfristrup/SoundScapes.
