# Supplementary material for: A stochastic simulation model for assessing the masking effects of road noise for wildlife, outdoor recreation, and bioacoustic monitoring
Source: Oecologia. 2022 May 6;199(1):217–28. doi: 10.1007/s00442-022-05171-2 (PMC9072761; doi:10.1007/s00442-022-05171-2)
Supplement: Supplementary file 2 — Supplementary file2 (DOCX 355 KB) [file 442_2022_5171_MOESM2_ESM.docx]

Soundscapes User Guide V.1.0.0

**Article**: *A stochastic simulation model for assessing the masking effects of road noise for wildlife, outdoor recreation, and bioacoustic monitoring*

**Journal**: Oecologia

**Authors:** Cory A. Toth^1^, Benjamin Pauli, Christopher J.W. McClure, Clinton D. Francis, Peter Newman, Jesse R. Barber^#^, Kurt Fristrup^2#^

**Corresponding authors:**

^1^ Department of Biological Sciences, Boise State University, Boise, ID 83725, USA; email: [tothcorya@gmail.com](mailto:tothcorya@gmail.com)

^2^ Natural Sounds and Night Skies Division, National Park Service, Fort Collins, CO 80525, USA; email: [Kurt.Fristrup@colostate.edu](mailto:Kurt.Fristrup@colostate.edu)

^#^Co-Principal Investigators

**Contents**

[1.](#_heading=h.gjdgxs) About Soundscapes 4

[1.1. Overview 4](#_heading=h.30j0zll)

[1.2. Purpose 4](#_heading=h.1fob9te)

[1.3. Entities, state variables, and scales 4](#_heading=h.3znysh7)

[1.3.1. Cells and virtual landscape 4](#_heading=h.2et92p0)

[1.3.2. Entities 5](#_heading=h.tyjcwt)

[1.3.3. Time 6](#_heading=h.1t3h5sf)

[1.4. Process overview and scheduling 6](#_heading=h.4d34og8)

[1.5. Design concepts 7](#_heading=h.17dp8vu)

[1.5.1. Basic principles 7](#_heading=h.3rdcrjn)

[1.5.2. Emergence 7](#_heading=h.26in1rg)

[1.5.3. Adaptation, Learning 8](#_heading=h.lnxbz9)

[1.5.4. Prediction 8](#_heading=h.35nkun2)

[1.5.5. Sensing 8](#_heading=h.1ksv4uv)

[1.5.6. Interaction 8](#_heading=h.44sinio)

[1.5.7. Stochasticity 9](#_heading=h.2jxsxqh)

[1.5.8. Collectives 9](#_heading=h.z337ya)

[1.5.9. Observation 9](#_heading=h.3j2qqm3)

[1.6. Initialization 10](#_heading=h.1y810tw)

[1.7. Input data 11](#_heading=h.2xcytpi)

[1.8. Submodels 12](#_heading=h.1ci93xb)

[1.8.1. Vehicle movements 12](#_heading=h.3whwml4)

[1.8.2. Vehicle noise output 12](#_heading=h.2bn6wsx)

[1.8.3. Noise propagation 12](#_heading=h.qsh70q)

[1.8.4. Searcher update and movement 13](#_heading=h.3as4poj)

[1.8.5. Acoustic detection 13](#_heading=h.1pxezwc)

[1.9. Limitations 14](#_heading=h.49x2ik5)

[2. Getting Started 14](#_heading=h.2p2csry)

[2.1. Installing NetLogo and opening Soundscapes 14](#_heading=h.147n2zr)

[2.2. The user interface 15](#_heading=h.3o7alnk)

[2.2.1. Inputs 15](#_heading=h.23ckvvd)

[2.2.2. Habitat view 16](#_heading=h.ihv636)

[2.2.3. Outputs 17](#_heading=h.32hioqz)

[2.2.4. Other 17](#_heading=h.1hmsyys)

[2.3. The code 18](#_heading=h.41mghml)

[3. Behavior Space simulations 18](#_heading=h.2grqrue)

[3.1. Setting up Behavior Space 18](#_heading=h.vx1227)

[4. References 21](#_heading=h.3fwokq0)

# About Soundscapes

## Overview

Soundscapes is a customisable, agent-based model that simulates the effects of road traffic noise on the ability of animals to perceive acoustic resources in an adjacent landscape. It also models the ability of human hikers and bioacoustic monitors to detect those same animals. Road noise affects acoustic detection by masking other sounds, reducing the distance at which sounds can be perceived. Traffic noise varies in space and time across the landscape as vehicles change their positions and speeds on the road. Calibrated vehicle source levels and ISO-9613 sound propagation procedures provide the capacity to connect model results to realistic traffic conditions.

## Purpose

The purpose of the Soundscapes model is to provide researchers and landscape managers with a tool to assess the effects of road traffic noise on the acoustic experience of animals and humans. The model simulates the behaviour of animals searching for acoustic resources (hereafter referred to as “searchers”) in a landscape adjacent to a road. These searchers experience varying sound levels that diminish the maximum range for detection of resources. Second, the model evaluates the diminished capacity of humans and acoustic instruments to detect searcher sounds due to noise.

## Entities, state variables, and scales

### Cells and virtual landscape

Square cells in this model are exclusively assigned to one of the following categories: **road**, **trail**, **resource**, or **background** matrix. The virtual landscape is 400 x 301 pixels with the origin at the center of the leftmost column. One pixel represents a 10 m square. Cells with a with an x-coordinate equal to zero are designated as a **road**. Cells with y-coordinates equal to zero are designated as a **trail**. The trail bisects the landscape but does not extend beyond it.

Several cells within the landscape are designated as **resources**. These are either distributed randomly or biased away from the road due to traffic noise. The number of resources is specified by the expected distance travelled by a searcher between resource encounters. Note that changing the ambient (noise-free) sound level alters the area searched per meter travelled, which alters the spatial density of resources unless a concomitant change in distance travelled between resources is made.

The cells in the model play a second role as locations on a virtual road. Conceptually, habitat patches are stacked column-wise, with a virtual size of 1 m square. The road is placed at x = 0. Y coordinates range between +/- 200*301 + 150 m (60.35 km). The patches on the virtual road that correspond to the orange line on the left edge of the ecological habitat are marked by pale orange area bracketing the central column (x = 200; Fig. 1). Vehicles that are seen within the pale orange area are currently travelling adjacent to the model’s landscape (i.e., the section of road denoted by the dark orange line at the model’s left edge). Columns to the left of this pale orange band represent road segments approaching the ecological habitat. Columns to the right represent departing road segments. Vehicles transiting beyond the top of one column reappear at the bottom of the column to the right. Vehicles exiting the top of the rightmost column reappear at the bottom of the leftmost column. At 60 km range this sudden shift of vehicles from departing to approaching will have negligible effect on spatial patterns of noise exposure. Functionally, this arrangement creates a road of indefinite acoustical length with a continuous stream of traffic.

Vehicles have a single state variable: speed. Searchers have two state variables that modulate behavior: moving to a resource and a list of unvisited resources detected.

### Entities

The entities in this model are **searchers**, **stations**, and **vehicles**.

A user-defined number of homogenous **searchers** are initially placed at random throughout the landscape. Searchers travel the landscape at a constant speed with random deviations in course when they are not approaching resources. Searchers keep a record of the resources they have detected during a simulation, including the resources they would have detected had noise been absent.

The number and fixed locations of listening **stations** can be changed in the code. For demonstration purposes, five stations are placed on patches at increasing distances – 50 m, 550 m, 1250 m, 2240 m, and 3590 m – away from the road along the trail. Stations keep a record of the number of searchers that pass within their noise-limited detection distance, as well as the number of searchers that would have been detected in the absence of noise.

Finally, a user-defined number and type of **vehicles** populate the road. The type of vehicle – either sedan or motorcycle – is dictated by the state variable *veh-name*, and is selected by the user prior to model run. Vehicles within a set are identical, yet the level and spectral shape of their noise output changes with speed. Vehicles are confined to move in one direction along the virtual road.

### Time

A single iteration in the model represents 1 s. Agent speeds are specified in meters moved per time step, or m/s.

## Process overview and scheduling

Searchers move during each time step with the intention of finding unobserved resources. The user defines the base perceptual range (maximum detection distance) of the searcher. The radius of the listening area (see [Design concepts](#_heading=h.17dp8vu) section) is calculated based on the sound level (see [Submodels](#_heading=h.1ci93xb) section). Sound levels are calculated based on the distances between pixels and every vehicle (and their speeds) at iteration of the model.

Searchers move at a constant speed with Gaussian deviations from their previous course until a resource falls within their listening area. This happens because they move toward the resource, the noise level falls, or both. When a resource is heard, the searcher orients towards the resource and moves without course deviations until they land on the patch. Then they move toward the next resource they heard (perfect memory) or resume searching along the course that took them to the resource (with deviations). When a searcher is moving towards a resource their color changes to lime green.

Listening area is calculated per timestep for the potential human listeners on the trail that bisects the landscape and the listening stations (which could be placed anywhere). The maximum listening area size is a multiple of the size of the searchers’ maximum listening area (see [Submodels](#_heading=h.1ci93xb) section). Noise decreases all listening areas by the same proportion. The model tracks the duration for which searchers were audible from the trail. When a searcher is audible from the trail, the colour of the nearest trail patch turns from yellow to blue. For listening stations, the model records all instances when searchers passed within the maximum range of detection and the masked detection distance. This enables direct calculation of the number of searchers missed due to noise.

The basic order of the processes in each time step are:

1. Increment the iteration counter (**tick**).
2. Update trail colors to fade towards the background color.
3. Move vehicles
4. Update searchers (noise exposure, birdability)
5. Move the searcher
6. Update stations (noise exposure, searcher detections)
7. Update the GUI displays.

## Design concepts

### Basic principles

Noise masks the perception of sounds by increasing the threshold required for detection and by reducing the listening area (Barber et al. 2010; Francis and Barber 2013). Listening area – the area within which a sound can be heard by a receiver – changes in response to sound levels. A 3 dB increase in sound level at the same frequency as a relevant signal reduces listening area by as much as 50% (see Barber et al. 2010). Fluctuations in listening area due to noise exposure are the pivotal feature of Soundscapes model. Noise degrades all auditory detection. Combining searcher and human listening performance creates opportunities to explore the coupled human-wildlife dynamics of interactions through sound (Francis et al. 2017).

The resources in our model produce acoustic signals. They can represent either individuals producing communication calls (e.g., male territorial or mating advertisement), or prey items producing adventitious sounds (e.g., Goerlitz et al. 2008). All such sounds are subject to masking by noise (e.g., Bee et al. 2001; Halfwerk et al. 2011; Siemers and Schaub 2011). The movement and acoustic detection performance of searchers can be adjusted to approximate the search behavior of many animal species.

The Soundscapes model approximates the detectability of the searchers by calculating how often they are within audible range of the trail bisecting the landscape. The base listening area of human observers and stations is set as a multiple of the base listening areas of the searchers using the *human-mult* variable set by the user.

Bioacoustic detectors and automatic recording units are increasingly popular instruments for surveys of wildlife populations and behavior. The Soundscapes model provides the capacity to evaluate the number of detections that an array of such equipment would record, given the density and movements of animals and the traffic noise regime.

Traffic noise transmission is affected by both atmospheric conditions (Ovenden et al. 2009). Atmospheric effects are incorporated into Soundscapes using a 1/3^rd^ octave band framework for vehicle noise propagation and ISO-9613 procedures to compute sound attenuation. A-weighted summations of ambient sound spectra and vehicle noise levels are used to compute the effect of masking.

### Emergence

The movements of vehicles on the road borrow concepts from the Simple Traffic Model in the NetLogo library. Parameter settings enable simulation of congestion and even stoppages on the road. These traffic patterns modulate noise exposures in the adjacent habitat. These complex patterns of noise exposure modulate acoustic detections of resources by searchers and searchers by humans. Searcher movements incorporate fewer abrupt turns as noise levels increase, due to reduced detections of resources. Behavioral responses to noise levels and interactions between vehicles and wildlife could be introduced to create new dimensions of emergent behavior.

### Adaptation, Learning

None of the agent behaviors change with experience or time.

### Prediction

Agents do not predict future conditions.

### Sensing

Searchers, human users, and stations sense the acoustic landscape within their listening area. Each searcher’s perceptual space is portrayed as a circle whose size shows the listening area and whose grayscale value is proportional to noise exposure in decibels (a logarithmic scale).

The audibility of searchers to humans along the path located is calculated by how often the searcher is within the masked detection distance of listeners on the path. Searchers also accumulate measures of how often they would have been audible from the path in the absence of noise. Listening stations accumulate measures of how many searchers they detected in the presence of noise, as well as how many they would have detected without noise.

Reductions in listening area via masking are calculated from the ratio of ambient to ambient + noise sound levels, using A-weighting to sum sound energy across 1/3^rd^ octave bands before calculating this masking coefficient.

When searchers hear sounds from a resource they localize them perfectly and move towards them without deviation.

### Interaction

Vehicles interact with other vehicles that they overtake. Otherwise, agents do not interact (though additional programming might implement responses to sounds beyond resource visitation).

### Stochasticity

The initial locations of resources and agents within the landscape is random. The direction of movement of searchers not moving towards a resource follow Gaussian deviations from their previous courses. Searchers encountering a boundary scatter forward from their present course with a random combination of specular reflection and an exit angle of 17 degrees. Vehicles have different maximum speeds, drawn from a gamma distribution. Vehicle accelerations at each iteration vary randomly from their shared, expected value.

### Collectives

Searchers have no collective behaviour. Vehicles can interact in ways that generate congestion and stoppages in traffic flow.

### Observation

Interactive reports of average distance travelled per resource encountered (in meters), fraction of searchers audible from the trail, median vehicle traffic rate, and mean vehicle speed are displayed. Average ticks per hour is displayed as a diagnostic predictor of simulation duration. A histogram of vehicles speeds is displayed to reveal the extent of slowdowns and stoppages.

Searchers and stations store extensive information for customized retrieval at the end of simulations. The term “birdable,” which applies to all taxa, refers to intervals when searches are within audible range of the trail.

| Searchers | Stations |
| --- | --- |
| Resources visited | Searchers detected |
| Resource detected if no noise | Searchers detected if noise were absent |
| History of sound exposure | History of sound exposure |
| Sound levels when resources detected | Sum of noise when no searchers detected |
| Sound levels when birdable | Detection durations |
| Birdable durations | Detection durations if noise were absent |
| Birdable durations if noise were absent | Absence durations |
| Absence durations | Absence durations if noise were absent |
| Absence durations if noise were absent |  |
| Distances from road when birdable |  |

## Initialization

Prior to starting a simulation, the user should review or select values for the variables that parameterize the model. When the *Setup* is pressed two sets of variables are initialized: graphical elements in the model display and embedded variables in the code.

Table 1: **Variables set through the graphical user interface**

| Meters-moved-per-resource | Sets resource density: the expected distance traveled in a straight line to encounter a resource. |
| --- | --- |
| Num-searchers | The number of wildlife agents. |
| Searcher-speed | The speed of wildlife agents, common to all. |
| Search-turn-sd | The standard deviation of Gaussian deviations from a searcher’s previous course (in degrees). |
| Ambient-level | The sound level in the environment when no noise is present (in decibels: dB). |
| Base-percept | The maximum distance at which a resource can be heard in the absence of noise (in meters). |
| Human-mult | The multiple of *base-percept* at which humans can hear searchers (in meters). |
| Num-vehicles | The number of vehicles on the road. |
| Speed-limit | The nominal limit of vehicle speeds; the expected value of maximum speed for each vehicle (in kilometers per hour). |
| Speed-limit-SD | The standard deviation of vehicle maximum speeds. |
| Acceleration | The constant acceleration experienced by each vehicle when they are not at their maximum speed or obstructed by a vehicle in front of them (in meters per second per second). |
| Excess-brake | When overtaking a vehicle ahead and not passing, the difference between the obstructing vehicle’s speed and the approaching vehicle’s speed realized after slowing (in kilometers per hour). |
| Vpass-probability | The probability of passing an obstructing vehicle without slowing down. |
| Height-noiz | The effective height of the vehicle noise source (in meters). |
| Height-rcvr | The effective height of the searchers, humans, and monitoring devices (in meters). |
| Temp-celsius | Air temperature (in degrees C). |
| Atm-pressure-bar | Atmospheric pressure (in bar). |
| Relative-humidity | Atmospheric RH (in percent). |
| Ground-hardness | A value between zero and one expressing the acoustic hardness of the ground. |

Table 2: **Variables embedded in the code**

| Ref-distance | The distance at which vehicle noise levels were specified. |
| --- | --- |
| Meters-per-unitNL | The distance between patch centers in the ecological space (in meters). |
| Num-ranges | Controls the resolution of attenuation and range-speed lookup matrices used to accelerate noise propagation computations. |
| Reflect-bias | Forward scattering of searchers encountering a boundary is randomly biased towards this value (specified as the arctangent of the desired angle). |
| Noise-decorrelate-ticks | Sets the sampling intervals for searcher and station sound level histories (in seconds). |
| Accel-jitter | Controls random fluctuations in vehicle accelerations. |
| Kolr-decay | Sets the fading rate for colored markers of searcher positions projected onto the trail. |
| Base-searcher-size | The size of searcher symbols in the absence of noise. |
| Freqs | The center frequencies of spectral elements used in noise propagation calculations (in Hertz). |
| aWt | The A-weighting factors applied to each spectral band before summation (converted from decibels to a multiplicative factor). |

In addition to initializing the variables embedded in the code, clicking the *Setup* button also creates a lookup table that precomputes the amount of noise received by a *searcher* or *station* based on the distance to the vehicle and the vehicle’s speed. It is much faster to interpolate received noise values between speed and distance values than it is to calculate source level and propagation loss at each iteration, especially because the propagation loss is computed for 24 spectral components.

## Input data

The model utilizes input data on vehicle noise output as a function of speed. These inputs are presently derived from calibrated, 1/3^rd^ octave band descriptions of vehicles in the NMSim source library. A small script written in R (R Core Team 2022) fits power law functions to measured noise levels at three speeds. The resulting coefficients are stored in CarCoef2.tsv and MotoCoef2.tsv.

## Submodels

We created submodels for the movements of vehicles, the generation of vehicle noise, the propagation of noise, the movements of searchers, and acoustic detection.

### Vehicle movements

Vehicles each have a maximum speed, which is equal to speed-limit on average, yet varies with a gamma distribution. At each iteration vehicles move forward at their present speed unless they are obstructed by a slower vehicle ahead of them. If a movement puts them above the upper boundary, they relocate to the next column at the same distance above the lower column. If they are in the last column, they relocate to the first column. Otherwise, a vehicle may pass the vehicle without slowing down with probability *vpass-probability* or move to a position two seconds behind the lead vehicle. When braking to follow another vehicle, the speed is equal to the lead vehicle’s speed minus *excess-brake*. After completing their movement, the vehicles increase their speed (for the next iteration) by *acceleration* adjusted up or down by a small random increment.

### Vehicle noise output

The vehicle type is selected prior to clicking *Setup* by choosing one of the drop-down options for the *veh-name* button. Each vehicle type has different noise output levels and spectra. At present the options are sedans and motorcycles. Motorcycles are about ten times as loud as sedans, and their spectrum is shifted towards lower frequencies. Lower frequency noise propagates with less atmospheric absorption. Sedan and motorcycle noise output and spectra were drawn from 1/3^rd^ octave band data packaged with the NMSim noise model (Downing 2004; Ikelheimer and Plotkin 2005). A small R script processed 1/3^rd^ octave band spectra at three speeds to fit a power-law function for each spectral band. The power-law coefficients are imported into NetLogo to predict noise band level as a function of speed. All vehicles of each type are identical to each other. The level and spectral shape of vehicle noise output changes with speed. Faster vehicles produce more noise. All vehicle noise measurements are calibrated at a fixed reference distance; *ref-distance* is specified by a global constant in the code (304.8).

### Noise propagation

In each iteration the noise received by each searcher or station from each vehicle is calculated from a lookup table that yields the A-weighted, pressure-squared noise level based on vehicle speed and the distance between the vehicle and the receiver. The sum of received noise from all vehicles is added to the ambient sound level, and then converted to a calibrated decibel value.

The receive noise level values in the lookup table are computed at when the user clicks the *Setup* button. These calculations apply ISO-9613 propagation procedures to 24 1/3^rd^ octave band center frequencies, returning multiplicative factors resulting from taking the antilog of the ISO-9613 outputs. A-weights, attenuation, and vehicles source levels are multiplied together for each 1/3^rd^ octave band before these products are summed and converted to a scalar decibel value.

### Searcher update and movement

After updating the received sound level, each *searcher* adjusts the size and shade of gray to express its listening area and level of noise exposure (darker is louder). Each searcher continues to move toward a resource without deviations if it had previously initiated such movement. Searchers moving towards a resource are colored lime green. Otherwise, if unvisited resources had been detected previously then the searcher orients towards the closest resource and moves toward it. If the searcher is not moving towards any resource, then it randomly alters its course and moves at *searcher-speed.*

During the search phase the edges of the landscape are impassable to searchers. When a searcher’s next move would place them outside the bounds of the simulation, their course is reflected forward from their present course with a randomized bias towards a 17 degree departure heading (relative to the boundary). This randomized departure procedure was found to reduce the reduction in the number of resources visited due to edge artifacts during model development. At the end of a search phase movement, the searcher adds any unvisited resources that are audible to its list for future visitation.

### Acoustic detection

The *base-percept* variable determines the maximum range of resource detection in the absence of noise. The *human-mult* variable sets the maximum range of searcher detection in the absence of noise, which is *base-percept * human-mult.* After sound levels are updated, each searcher or station calculates the current *percept* as a fraction of the *base-percept* using the following formula:

*Equation 1*

$$percept=basepercept* {10}^{\left( \frac{\left( sound-ambient \right)}{10} \right)}$$

All unvisited resources within the perceptual range of the searcher are added to its list for future visitation. All searchers within human detection range of the path add to their internal record of birdability. All searchers within human detection range of a station are added to that station’s detection record. Stations change color when from grey to sky blue when searchers are in range. For resources, birdability, and station detections, potential detections are logged as they would have been in the absence of noise.

## Limitations

Users should be aware of several simplifications that constrain interpretations of model results. A-weighted summations of sound spectra are widely used to evaluate noise effects on both humans and wildlife, though the weightings are derived from human perceived loudness curves. Even in humans, A-weighted calculations are not the best method for evaluating the audibility of a sound. The 1/3rd octave band framework could support more sophisticated models of sound detection.

The model presently assumes that the sounds produced by resources and searchers will be masked in proportion to the increase in A-weighted sound level caused by traffic noise. This implies that all listeners are operating in a masked detection regime: they are not limited by their intrinsic auditory thresholds. Furthermore, this implies that spectral separation between signals of interest and noise does not diminish the ratiometric effect of increasing A-weighted sound levels. Also, the model does not account for other auditory processing that can reduce masking (e. g. comodulation release). Noise may interfere with auditory perception by mechanisms other than masking, including distraction and decreased auditory attention.

Absorption and scattering by vegetation (Bucur 2007) is not modeled. These effects are generally small in comparison with divergence and atmospheric absorptive losses.

One height is used for all receivers (*searchers* and *stations*), set by *height-rcvr.*

Increased engine noise during vehicle acceleration is not considered.

# Getting Started

## Installing NetLogo and opening Soundscapes

NetLogo was developed by Wilensky (1999) and serves as an open-source, multi-agent, programmable modeling environment. NetLogo may be downloaded for free from <https://ccl.northwestern.edu/netlogo/download.shtml>. Detailed user instructions for its installation and operation may be found at this link: <https://ccl.northwestern.edu/netlogo/docs/>.

Double-clicking the Soundscapes icon will open the program once NetLogo has been downloaded and installed.

## The user interface

The user will be presented with the main interface upon opening Soundscapes.


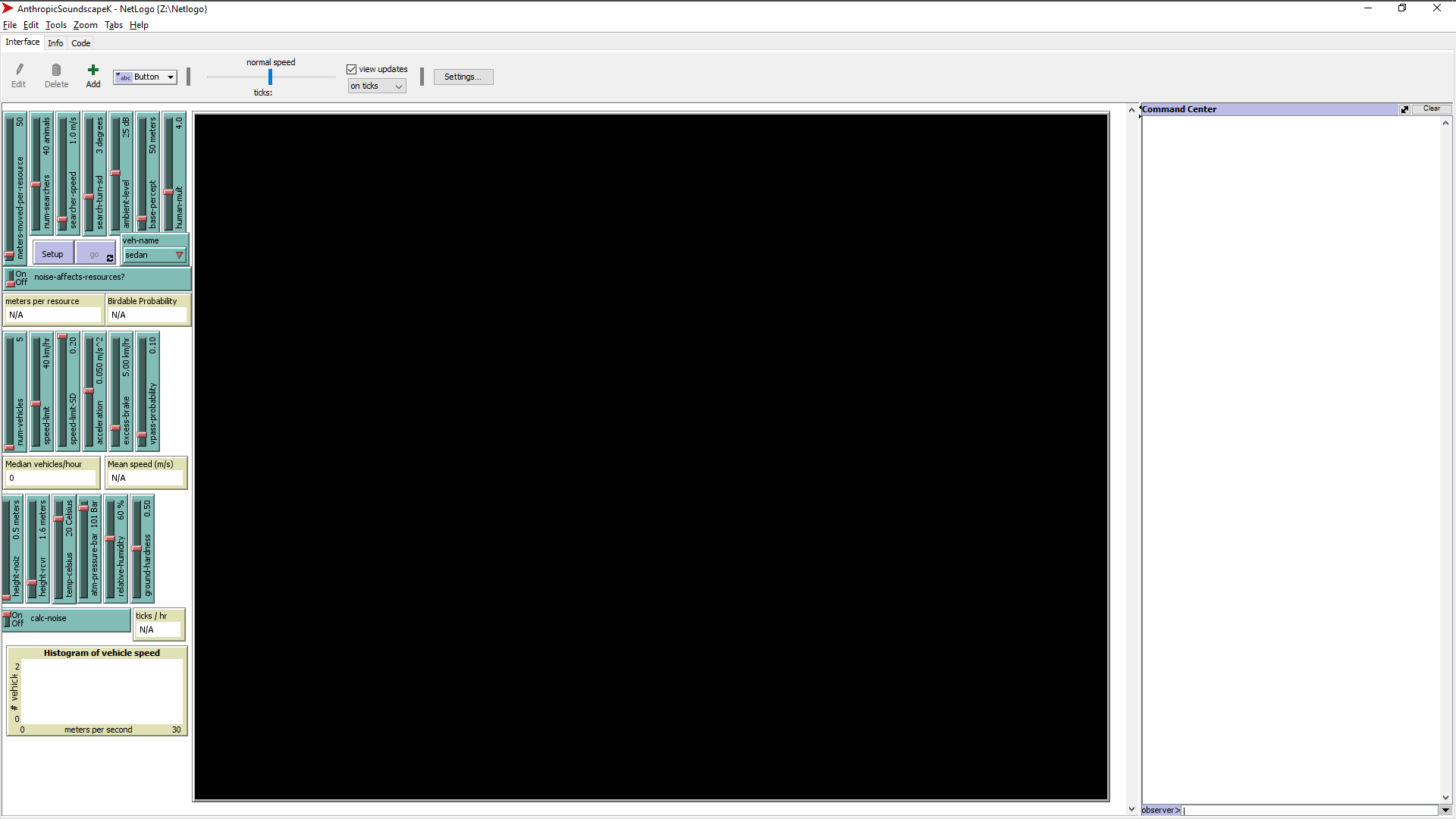


*Figure 1: Screen capture of the Soundscapes model display prior to setting up a simulation. The interface consists of two buttons (Setup, Go), (green switches, sliders, and dropdowns), outputs (beige frames), and the habitat view (black prior to clicking Setup).*

### Inputs

The input selections to the left of the habitat frame are organized into three rows: searcher and environmental settings, vehicle settings, and acoustical physics settings. Iteration speed becomes slower as you increase the number of *searchers*, the number of *vehicles*, and the number of *stations*. Noise propagation is calculated between each *vehicle* and the *searchers* and *stations*.

- **Setup** will initialize a model run based on the user-inputted settings
- **Go** runs the model indefinitely. You can edit the **Go** button to deselect the *Forever* box. Subsequently, clicking the **Go** button will just execute one iteration.

### Habitat view

In the centre of the interface is the world view. The view will be blank prior to model initialisation. Hitting the **setup** button will initialise the model based on the user-defined inputs:


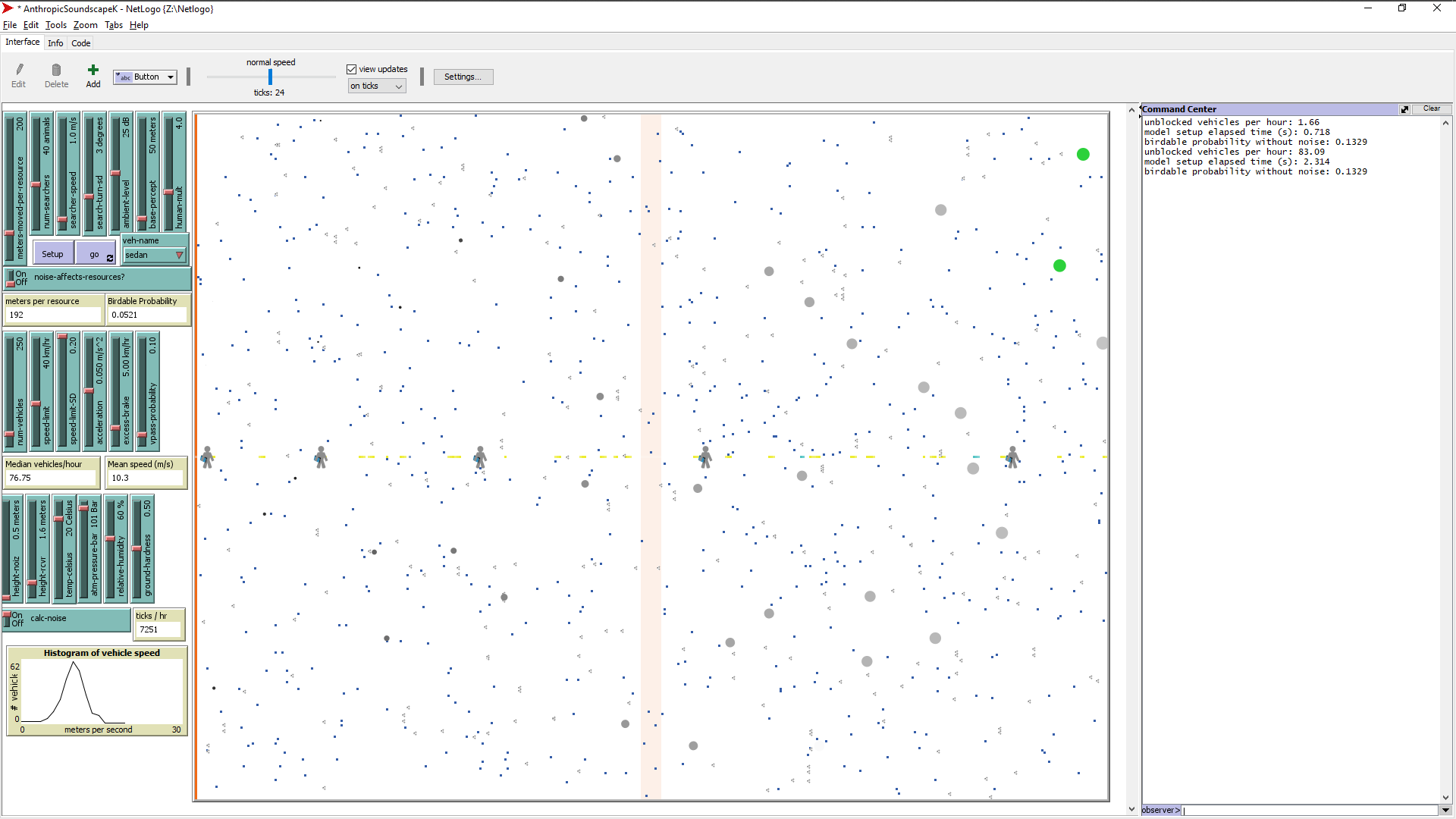


*Figure 2: Habitat view with resources (blue dots), road (orange line, left side), virtual road area (pale orange area in the central columns), central trail (intermittently colored yellow and blue pixels, displaying the history of x-coordinates for searchers).*

Note that the ecological habitat is 400 x 301 pixels in size, which corresponds to a landscape that is 4 km x 3.1 km.

### Outputs


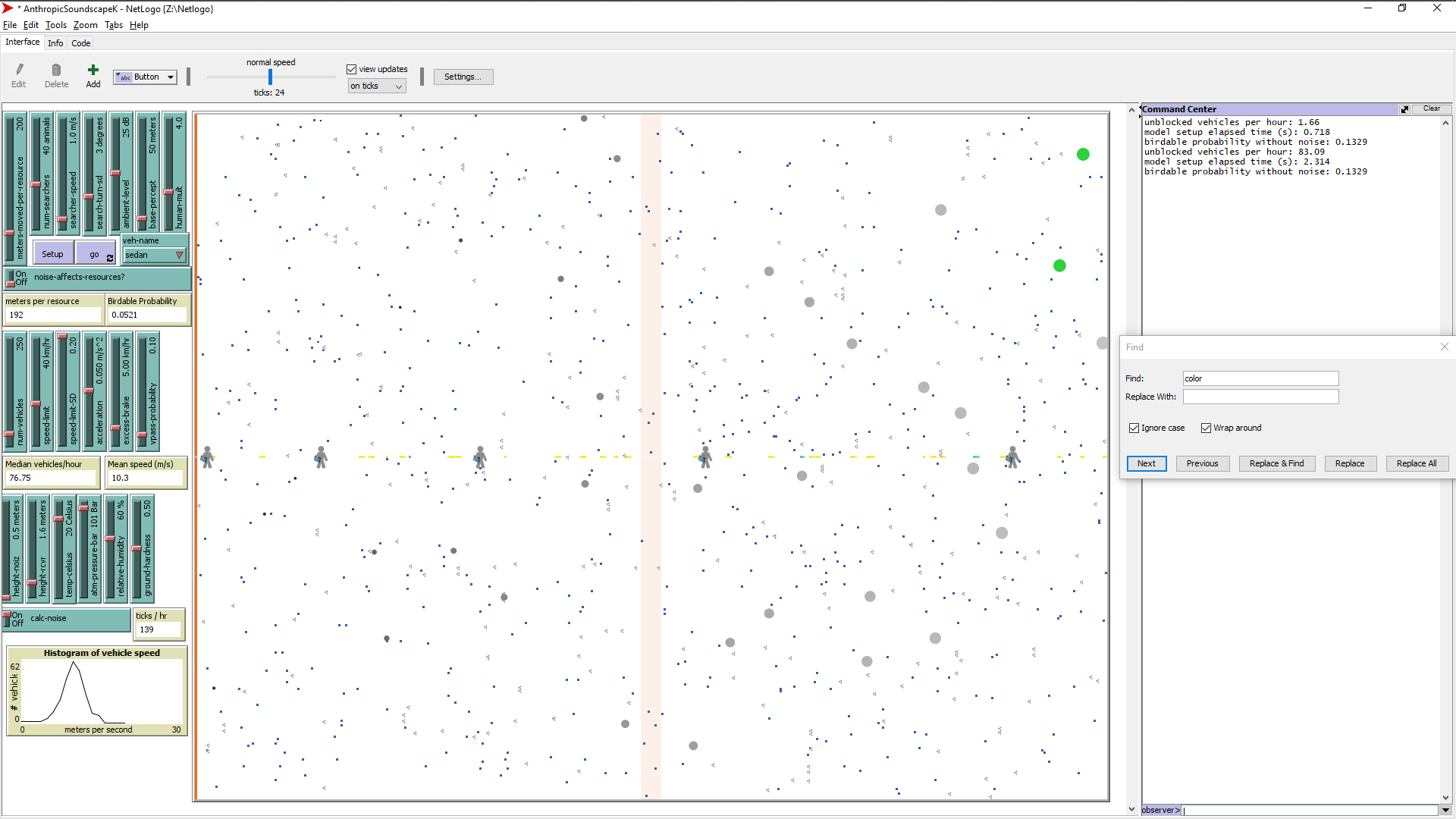


*Figure 3: Model status indicators (tan boxes). Noise-limited searcher success, searcher availability to birders, traffic density, traffic speed, simulation speed, and distribution of vehicle speeds.*

### Other


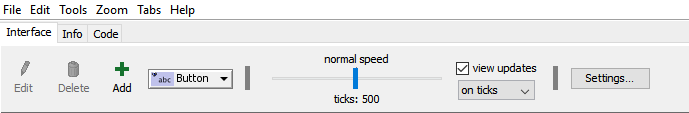


Along the top of the program are a number of settings that affect the way the model looks and behaves. This includes the ability to Edit, Delete, or Add interface elements, such as buttons, input components, or output plots. The slider in the centre determines the speed at which the model will advance through timesteps. Selecting “view updates” will determine if the world view will be updated during model run (doing so will slow down model run), and the dropdown determines if the habitat view is updated at with each timestep or continuously. Clicking the settings button will adjust components of the habitat view, including its size.

## The code


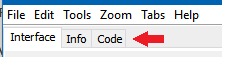


Clicking on the Code tab near the top of the program will show the underlying model code. The code can be inspected to explore details of how the agents behave, how noise is propagated, and the information stored by agents. Programmers can modify the code to adapt the model to their interests and to add new features.

# Behavior Space simulations

While it can be entertaining and educational to observe Soundscapes’ dynamics as an animation, systematic exploration of alternative scenarios will require use of the Behavior Space tool in NetLogo. Behavior Space sets up a collection of scenarios for simulations by choosing ranges for the input variables. Outputs that summarize the results of each simulation are specified by the tool, which can save these results as either a table or spreadsheet format. Consult the NetLogo User Manual for distinctions between these output formats.

## Setting up Behavior Space

Selecting BehaviorSpace under the Tools menu will allow users to execute a batch of simulations.
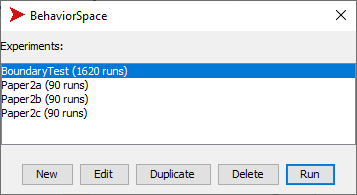


Selecting Edit on the Paper2a Scenario will bring up the following dialog:


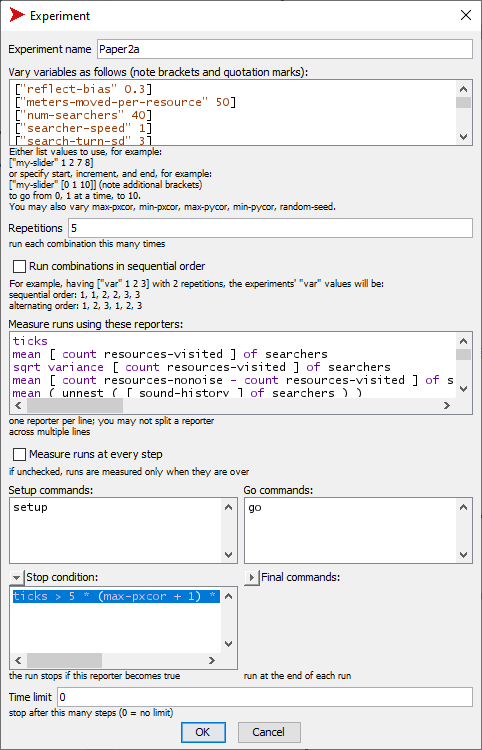


Note that the entries in “Measure runs using these reporters:” box are NetLogo commands that can be tested in the Command Center on the main NetLogo display. It is advisable to test every reporter before you execute a Behavior Space batch process.

Once the scenario is ready, select Run, and the following dialog box will appear:


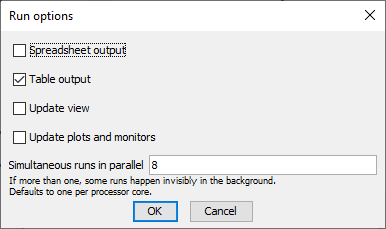


The selected box options deliver the fastest simulation speeds. “Simultaneous runs in parallel” can be set to the maximum number of cores on your machine for fastest execution.

# References

Barber JR, Crooks KR, Fristrup KM (2010). The costs of chronic noise exposure for terrestrial organisms. Trends Ecol Evol 25:180-189

Bee MA, Kozich CE, Blackwell KJ, Gerhardt HC (2001). Individual variation in advertisement calls of territorial male green frogs, Rana clamitans: implications for individual discrimination. Ethology 107:65-84

Bucur V (2007). Urban Forest Acoustics. Springer-Verlag, Berlin, Heidelberg

Downing JM (2004). Noise simulation modeling for airport noise analysis, INTER-NOISE and NOISE-CON Congress and Conference Proceedings. Institute of Noise Control Engineering pp 2834-2841

Francis CD, Newman P, Taff BD, White C, Monz CA, Levenhagen M, Petrelli AR, Abbott LC, Newton J, Burson S, Cooper CB, Fristrup KM, McClure CJW, Mennitt D, Giamellaro M, Barber JR (2017). Acoustic environments matter: Synergistic benefits to humans and ecological communities. J Environ Manage 203:245-254

Francis CD, Barber JR (2013). A framework for understanding noise impacts on wildlife: an urgent conservation priority. Front Ecol Environ 11:305-313

Goerlitz HR, Greif S, Siemers BM (2008). Cues for acoustic detection of prey: insect rustling sounds and the influence of walking substrate. J Exp Biol 211:2799-2806

Halfwerk W, Holleman LJM, Lessells CK, Slabbekoorn H (2011). Negative impact of traffic noise on avian reproductive success. J Appl Ecol 48:210-219

Ikelheimer B, Plotkin K (2005). Noise Model Simulation (NMSim) User Manual, Wyle Laboratories Report WR 03, 9

Ovenden NC, Shaffer SR, Fernando HJS (2009). Impact of meteorological conditions on noise propagation from freeway corridors. J Acoust Soc Am 126:25-35

R Core Team (2022). R: A language and environment for statistical computing. R Foundation for Statistical Computing, Vienna, Austria

Siemers BM, Schaub A (2011). Hunting at the highway: traffic noise reduces foraging efficiency in acoustic predators. Proc Biol Sci 278:1646-1652

Wilensky U (1999). NetLogo. Northwestern University
